# The development of early warning scores or alerting systems for the prediction of adverse events in psychiatric patients: a scoping review

**DOI:** 10.1186/s12888-024-06052-z

**Published:** 2024-10-28

**Authors:** Valentina Tamayo Velasquez, Justine Chang, Andrea Waddell

**Affiliations:** 1 Institute of Medical Science, Temerty Faculty of Medicine, University of Toronto, Toronto, ON Canada; 2 Temerty Faculty of Medicine, University of Toronto, Toronto, ON Canada; 3 Waypoint Research Institute, Waypoint Centre for Mental Health Care, Penetanguishene, ON Canada

**Keywords:** Adverse events, Psychiatric patients, Predictive tools, Alerting system, Early warning

## Abstract

**Background:**

Adverse events in psychiatric settings present ongoing challenges for both patients and staff. Despite advances in psychiatric interventions and treatments, research on early warning scores and tools to predict patient deterioration is limited. This review provides a summary of the few tools that have been developed in a psychiatric setting, comparing machine learning (ML) and nonmachine learning/traditional methodologies. The outcomes of interest include the selected key variables that contribute to adverse events and the performance and validation measures of the predictive models.

**Methods:**

Three databases, Ovid MEDLINE, PsycINFO, and Embase, were searched between February 2023 and April 2023 to identify all relevant studies that included a combination of (and were not limited to) the following search terms: "Early warning," "Alerting tool," and "Psychiatry". Peer-reviewed primary research publications were included without imposing any date restrictions. A total of 1,193 studies were screened. A total of 9 studies met the inclusion and exclusion criteria and were included in this review. The PICOS model, the Joanna Briggs Institute (JBI) Reviewer's Manual, and PRISMA guidelines were applied.

**Results:**

This review identified nine studies that developed predictive models for adverse events in psychiatric settings. Encompassing 41,566 participants across studies that used both ML and non-ML algorithmic approaches, performance metrics, primarily AUC ROC, varied among studies between 0.62 and 0.95. The best performing model that had also been validated was the random forest (RF) ML model, with a score of 0.87 and a high sensitivity of 74% and a specificity of 88%.

**Conclusion:**

Currently, few predictive models have been developed for adverse events and patient deterioration in psychiatric settings. The findings of this review suggest that the use of ML and non-ML algorithms show moderate to good performance in predicting adverse events at the hospitals/units where the tool was developed. Understanding these models and the methodology of the studies is crucial for enhancing patient care as well as staff and patient safety research. Further research on the development and implementation of predictive tools in psychiatry should be carried out to assess the feasibility and efficacy of the tool in psychiatric patients.

**Supplementary Information:**

The online version contains supplementary material available at 10.1186/s12888-024-06052-z.

## Background

In recent years, quality improvement (QI) and safety has gained attention in several medical fields and more specifically, in mental healthcare. With patient safety incidents being the third leading cause of death in Canada [1], QI and safety has been a growing area in research to improve patient outcomes and enhance safety in healthcare settings [2]. However, like any medical field, mental health and psychiatry are not without its challenges. A critical challenge that remains in psychiatry is the complexity of the disorders and the difficulty this brings when assessing patients for risks. Part of QI and safety research in mental health involves targeting the causes that lead to high rates of safety-related incidents. In psychiatry, the prediction of risks, specifically adverse events, is an area to target, as earlier identification of a potential risk allows for earlier intervention and preventative measures to be taken.

### Adverse events in psychiatry

Improving patient safety is a central element in patient-centered medicine. An adverse event is generally classified as any noxious or unintended event occurring in association with medical care [3]. Adverse events can occur throughout the course of an inpatient stay, regardless of treatment or intervention.

The well-being and safety of patients and staff are always primary concerns in all areas of healthcare, including psychiatry. While adverse events are far more prevalent in psychiatry and mental health settings, the study of safety and quality improvement in these settings is more sparse than the rest of medicine [2]. Mental health settings include unique adverse event occurrences such as restraint, seclusion, self-harm, harm to others, and suicide [4]. Violence toward staff is one type of adverse event that may result in an injury to staff members and/or patients. A survey completed by staff at a psychiatric hospital revealed that 69.5% of staff had experienced violence from patients in the past year [5]. Adverse events included restraint and seclusion for patients. Restraint and seclusions are measures taken in psychiatric healthcare settings to manage patients who pose a risk to themselves or others [6]. These types of events can be extremely distressing and potentially harmful for patients and their physical and psychological well-being [7]. One Canadian study demonstrated that of the patients hospitalized at a psychiatric hospital in Montreal, 23.2% were secluded (with or without restraint), and 17.5% were secluded and restrained [8]. Identifying and managing adverse events promptly is crucial to prevent further consequences and harm as well as to ensure the overall safety of patients and staff in psychiatric settings.

Prevention of adverse events requires thorough accounting and investigation of the types of adverse events (AEs) that occur. There are several types of AEs unique to the field of psychiatry and mental healthcare, which may include but are not limited to seclusion, use of restraint, self-harm, suicidal behavior, patient violence and aggression, and staff harm [9]. The prevalence of a violence incident during admission averaged approximately 17% over 35 different facilities [10].

### Prediction models, risk assessments, and early warning systems

While routine inspection of AEs (through voluntary incident reporting systems or chart audits using “trigger tool methodology”) are necessary, they are not sufficient at preventing AE occurrences on their own. Trigger tools are methods to identify adverse events and tracking the rate of adverse events over time [11]. However, tools such as early warning systems are needed for adverse event prevention via earlier interventions. Early warning systems, a type of prediction model, are able to identify high-risk patients who are likely to deteriorate.

Patient deterioration is generally detected or predicted by clinical observation, vital signs, and structured risk assessment tools developed using statistical models. Examples of such risk assessment tools include the Broset Violence Checklist (BVC) and the Dynamic Appraisal of Situational Aggression (DASA) tool. Both BVC and DASA are actuarial risk assessments based on one-time assessments of risk factors, predict risk only in the very short term, and require frequent administration by clinical staff [12]. Nevertheless, the current screening tools and current methods for predicting patient deterioration in psychiatry patients are associated with their own limitations that still need to be addressed and improved. The literature indicates that these tools are not always effective in accurately predicting workplace violence incidents and events, highlighting the need for improved methodologies in risk assessment within psychiatric settings [13]. Another limitation of traditional psychiatric risk assessment tools is their reliance on manual completion by healthcare professionals. ML predictive models offer the potential for automated risk assessment by analyzing a large number of variables from the EMR of patient information to identify patterns and predict workplace violence events [14, 15].

Furthermore, other early warning systems tools that predict patient deterioration have been implemented in other areas of healthcare to prevent negative outcomes, such as in intensive care units (ICUs) [16, 17]. The National Early Warning Score (NEWS), a tool developed by the ​​Royal College of Physicians in 2012, is an example of an alerting tool that helps prevent further clinical deterioration in acute care settings [18]. NEWS2, an updated version, has become standardized and commonly implemented in different areas of the world [19]. Therefore, there is value and importance in using early warning systems to predict psychiatric deterioration [20].

### Machine learning

The occurrence of AEs is complex and influenced by “static” and “dynamic” risk factors. Using “patient violence” as an example of an AE, static (stable over time) risk factors may include age, gender, family history, traumatic experiences, or offenses during childhood [9]. Dynamic risk factors can fluctuate and may include psychiatric symptoms, substance use, and treatment adherence [9]. The Violence Risk Appraisal Guide Revised (VRAG-R) is a 12-item scored actuarial risk assessment tool to predict violence and re-offending risk in forensic psychiatric patients [21]. Although structured risk assessment tools such as the Violence Risk Appraisal Guide have shown acceptable predictive validity for predicting violent behavior, one of the disadvantages is the lengthy administration time.

The emergence of artificial intelligence (AI) has brought excitement for its potential to predict events more accurately, objectively, and faster. Machine learning (ML) is the system through which AI ‘teaches’ technology human-like intelligence. In the clinical setting, ML models, much like non-ML algorithms, have demonstrated the ability to predict outcomes from complex hierarchical data such as those contained in electronic medical records (EMRs) [22]. An example of a ML-predictive model used in general internal medicine is CHARTwatch. The CHARTwatch tool was developed using > 100 measurements recorded in routine practice and was first deployed in 2020 maintaining a performance of AUC = 0.76 after the first year of deployment [23]. As such, ML models may provide an additional layer of information to assist with clinical decision making in psychiatry [24].

In psychiatry, applications of machine learning or AI have not been as abundant as other imaging-heavy specialties. To the best of our knowledge, risk prediction tools based on ML for the detection of risk of adverse events in psychiatry wards are sparse. In recent years, some organizations have taken interest in developing similar models and tools in psychiatry. This review aims to identify existing risk prediction tools that predict AE in psychiatry wards using ML or other models, such as traditional statistics. This review will also describe their predictive performance, safety, and feasibility. Machine learning has emerged as a promising approach for predicting AEs in various medical fields. In the context of psychiatry, ML-based risk prediction tools have the potential to enhance patient care and increase safety for both patients and staff. The primary objective of this review is to identify and outline the early warning systems (both ML-based models and models using traditional statistics) developed for predicting AEs in psychiatric patients. This review will focus on summarizing the efficacy (predictive performance), safety, and feasibility of these tools in a psychiatric setting. By evaluating the available literature, this study aims to shed light on the current state of risk prediction tools in psychiatry and provide insights into their potential benefits and limitations.

## Methods/literature search

### Search strategy

The search was carried out in three electronic databases, Ovid MEDLINE, PsycINFO, and Embase, from February 2023 to March 2023 by applying the following search strategy: a combination of the following search terms: “Hospital Rapid Response Team”/or "Hospital Information Systems", “Alerting tool”, “Alerting signal”, “Early warning score”, “Incident reporting system”, “Incident monitoring system”, “Early warning signal”, “Psychiatry”, “Mental Health”, “Deterioration”, and “Patient”.

All the articles selected were peer-reviewed primary research publications. No restrictions were applied to the publication date. After carrying out the search strategy and importing articles to Covidence, the exclusion and inclusion criteria were applied to select articles to include in the review. The PICOS model, the Joanna Briggs Institute (JBI) Reviewer's Manual, and PRISMA guidelines were applied [25, 26].

### Inclusion/exclusion criteria

The PICOS framework was applied to create the inclusion and exclusion criteria outlined below:

1. Population: Patients with a mental health or psychiatric diagnosis or presentation admitted to psychiatric, mental health, or forensic settings. There were no age restrictions. Adult, adolescent, and pediatric patients were included.
2. Intervention: A digital/computerized prediction model risk that predicts the risk of adverse events or patient deterioration. Adverse events may include any noxious event to the patient, such as seclusion, use of restraint, self-harm, suicidal behavior, and patient violence toward other patients or staff.
3. Comparator: There were no comparison groups.
4. Outcome(s):
  a Measures of predictive performance (e.g., positive and negative predictive values)
  b Factors and variables included in the models.
5. Study Design:
  a The included primary articles, prospective studies, data linkage studies, and retrospective studies that included predictive performance measures
  b Non-English studies, case studies, secondary studies (reviews), abstracts, commentaries, editorials, opinion pieces, and animal studies were excluded.

### Study selection and data extraction

**Fig. 1:**
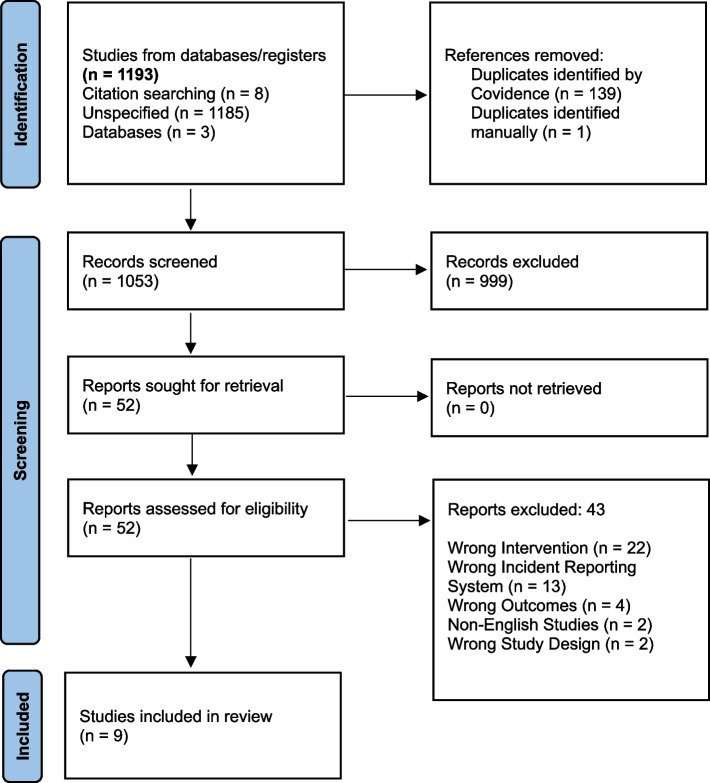
PRISMA Search Strategy Diagram. *From: search strategy diagram. On* Page MJ, McKenzie JE, Bossuyt PM, Boutron I, Hoffmann TC, Mulrow CD, et al. The PRISMA 2020 statement: an updated guideline for reporting systematic reviews. BMJ 2021;372:n71. https://doi.org/10.1136/bmj.n71

The screening process was conducted using two online tools, EndNote and Covidence. The references were imported into EndNote (see Fig. 1). A total of 1,193 studies were imported to Covidence and screened by article title, abstract, and full-text screening by two raters (JC, VT), and all conflicts were resolved by a third rater, AEW. The articles whose titles and abstracts were relevant underwent full-text screening. Then, articles that met the exclusion and inclusion criteria and parameters were included (*n* = 9). The following information was extracted from Table 1: study design, study setting (including country), sample size, population diagnosis, type of adverse event/outcome, patient characteristics (including sex and primary diagnosis), average age, and type of risk prediction model (ML or non-ML). The information in Table 2 includes the following: the predicted outcomes; the data source; the selected early warning model; whether the model was validated or not; the performance of the selected model; the validity of the selected model; the selected predictor variables; and the limitations. The extracted information in Table 3 includes the selected model (best-performing model) of each study and the variables included in the selected model. For all Tables, see Additional File 1.

## Results

### Study selection

The PRISMA review selection process for the articles included in the review is shown in the diagram in Fig. 1 [25]. A total of 1,193 articles were generated from the search strategy from both databases after EndNote removed duplicates. Using Covidence to screen for relevant articles from the title and abstract, 54 articles were selected. Finally, after the full-text screening phase, 9 articles met the inclusion criteria and were eligible for inclusion in this review.

### Study characteristics

From the 1,193 articles retrieved using the search strategy, 54 articles were eligible for full screening, 9 studies (*n* = 9) were included in this review, and the data from these studies were extracted. There was a total of 41,566 participants (N), and their data were included across the 9 studies. Some studies used the number of patients as their sample size, while other studies used the number of admissions (one patient can have multiple admissions) as their unit of sample size. The number of independent patients included in each study ranged from 89 to 29,841. Among the studies that reported patient age, the ages ranged from 28 to 59 years. Eight out of nine studies collected patient sex data, and across those studies, the subjects were predominantly male (64.7%). This included one study that included only male subjects. Patients included in the studies were adults with or without a preexisting psychiatric diagnosis admitted to a psychiatric or mental health institution. Geographically, the studies spanned multiple countries, including Denmark, the United Kingdom, the United States, China, and the Netherlands.

Of the studies included in this review, eight were retrospective studies, and one was a prospective study. The patient population was limited to individuals who were admitted to a psychiatric or mental health facility. The sample population mainly comprised adult patients at psychiatric units/institutions. The patients admitted to the different institutions across all nine studies varied in terms of their diagnoses, with some patients never having been diagnosed with a psychiatric disorder prior to admission.

The full details and a summary of the study characteristics and extracted data are provided in Table 1 (see Additional File 1).

### Primary outcomes: early warning systems and predictive models

This review included nine studies that investigated the development of prediction models for the risk of adverse events in psychiatric settings. Each study investigated different adverse events, ranging from mechanical restraints to violent offenses and emergent medical events. The risk prediction models used in these studies can be categorized into ML and non-ML approaches. The ML techniques include supervised machine learning algorithms [2], deep learning models [27], language processing models [27–30], least absolute shrinkage and selection operator (LASSO) regression [28], and support vector machines [28]. On the other hand, non-ML approaches include variations in multivariate/multivariable regression algorithms.

The primary outcomes of the reviewed studies included the performance of the prediction models developed in these studies. Most studies assessed performance by measuring the area under the ROC curve (AUC), the primary metric that allows us to assess the models. The ROC curve is a probability curve that is plotted with true positive rate on the y-axis and false positive rate on the x-axis. AUC is the value of separability between classes or categories. Therefore, a higher AUC indicates high performance and more accuracy in distinguishing between categories, and thus, high predictive value. However, it is important to note that all the models assessed here are very different.

 Danielsen et al., [31] demonstrated robust performance with an AUC of 0.87 with the random forest algorithm [31]. Furthermore, they validated the model with an independent portion of the dataset. At 94% specificity, they achieved a sensitivity of 56%, and the PPV was 8.1%. The only other study that achieved a greater AUC was that of Gou et al., 2021 [28]. This study developed three versions of their models, a sociodemographic and clinical model, a 3-way neuroimaging data model, and a model that combined the neuroimaging and sociodemographic data, with respective AUCs of 0.91, 0.91, and 0.95, respectively. However, they did not validate their models. Three other studies that validated their models achieved moderate performance. According to Menger et al., the 2019 AUC at site 1 was 0.79, and at site 2, it was 0.76. They cross-validated the models at both sites. The model developed at site 1 achieved an AUC of 0.72 at site 2 [29]. The model developed at site 2 achieved an AUC of 0.64 at site 1. Suchting et al., 2018 trained multiple models, including random forest gradient boosting machines and deep neural networks, but selected the best performing model, penalized generalized linear modeling (GLM) [30]. The GLM achieved an AUC of 0.78. After fivefold validation using other algorithms, they achieved an AUC of 0.78. Wang et al., 2020 also trained multiple models, including the binary logistic regression model, the least absolute shrinkage and selection operator, the elastic net, the gradient boosted regression trees, and the support vector machine classifier, and they selected the best performing model, the random forest model [32]. The random forest model achieved an AUC of 0.62. They used a fivefold validation technique. Finally, Menger et al., 2018 used a recurrent neural network model that achieved an AUC of 0.78 [27]. They did not validate this model.

Similarly, the performance of the non-ML models was also measured by the AUC. Using a multilevel logistic regression model, Fazel et al., 2021 achieved an AUC of 0.77 [12]. This study did not validate our findings. Graytak et al., 2017 reported an AUC of 0.91 for a multiple logistic regression model that used 12 variables and an AUC of 0.76 for the same model but with 6 variables [33]. Moreover, Geiss et al., 2018, was the only study that did not report an AUC measure of performance [34]. This study selected a logistic regression model, and although they did not use the AUC as a measure of performance, they reported the odds ratio for three factors retained in the selected model as well as the specificity of the tool.

Details of the primary outcomes are provided in Table 2 (see Additional File 1).

### Secondary outcomes: selected variables and factors for the predictive models

In the studies included in this review of early warning systems, there was diversity in the variables and factors selected as the predictive variables to include. While the studies were able to develop various models with various predictive variables, some common variables identified across multiple studies included admission type, age, sex, sociodemographic variables, behavioral factors, patient medical history, and clinical text variables. A unique variable included in one of the models in a study was neuroimaging data.

### Admission type

The identification of admission type (voluntary or involuntary), as a key predictor, suggests that the circumstances in which an individual was admitted may play a role in predicting the patients’ mental health outcomes and future incidents [31]. These findings could reflect the influence of the nature of the psychiatric crisis leading up to admission on the patient’s subsequent experience and outcomes.

### Behavioral factors and patient history

The significance of past medical history and previous behavioral incidents may underscore a relationship between previous events and future incidents and adverse events. For instance, nonadherence to therapy or medication [12], aggressive behavior, both verbal and physical [12, 28], substance misuse [32, 33], history of abuse both as a perpetrator and witness [30], and suicidal ideation [31, 32] are all examples of common variables included in multiple predictive models. Focusing, understanding, and considering the cumulative impact of such factors may contribute to a more comprehensive predictive model.

### Clinical text variables

The use of clinical text data [27, 29] highlights the rich information embedded in routine clinical practice. Natural language processing techniques have the potential to help extract these types of variables and provide insight from textual data.

### Age, sex, and sociodemographic variables

Age, sex, and sociodemographic variables are the most common variables selected for various models.

Details of the secondary outcomes are provided in Table 3 (see Additional File 1).

## Discussion

This review of the development of early warning systems in psychiatric settings offers valuable insight into the methodology, utility, accuracy, and diverse landscape of methodologies and their corresponding outcomes. The next section will focus on addressing the differences in performance across predictive tools used in other areas of healthcare.

### Performance across outcomes

The model that resulted in the highest AUC was the random forest (RF) ML model by [31], with a score of 0.87 and a high sensitivity of 74% and a specificity of 88% [31]. The area under the curve (AUC) is a measure used to evaluate the performance, generally, of a classification model. It is derived from the receiver operating characteristic (ROC) curve, which is a graph representing a model’s ability to discriminate between positive and negative classes. In all but one of the studies included in this review, it was used as a measure of performance, allowing us to compare the performance of all the models developed.

Compared with those of previous early warning predictive models developed for other areas of healthcare, the area under the ROC curve (AUC) of the nine models developed for psychiatric settings are similar to those developed for other areas in healthcare. For instance, the ‘SWIFT score’ is a predictive tool developed in the USA from a multivariate model to assess patients in ICUs. The tool resulted in AUC ROC scores of 0.75 and 0.70, respectively. In another example, a recent systematic review compiled and compared the use of four predictive tools for sepsis diagnosis and adverse outcomes, showing the importance of such tools for effective resource allocation and earlier interventions [17]. The four tools summarized and compared were the National Early Warning Score (NEWS), the Quick Sepsis-related Organ Failure Assessment (qSOFA), SIRS: Systemic Inflammatory Response Syndrome (SIRS), and the Sequential Organ Failure Assessment (SOFA). The AUROC scores for these tools were 0.71, 0.81, 0.61, and 0.71, respectively. These scores closely resemble the scores found in the nine studies outlined in this scoping review (see Table 2) (see Additional File 1).

### Selected variables

The importance of proceeding with a multifactorial approach is crucial to the development of a prediction tool. The inclusion of diverse variables, including demographic and clinical characteristics, behavioral elements, and even neuroimaging factors, reflects the complexity of mental health and psychiatric disorders. Therefore, by testing a large group of variables and selecting the most predictive ones to retain for the predictive model, the models can be trained to capture the patterns and weights of different variables with a moderate level of accuracy. A recent review highlighted the importance of using predictive variables and tools to detect psychiatric deterioration as well as its potential to facilitate patient assessments [20].

### Implications for clinical practice

The studies included in the review highlight the opportunity for future predictive models to be implemented and adopted in clinical practice as support for healthcare providers. The findings prove an advancement in the range of ways to include predictive methodologies in healthcare, specifically mental health and psychiatric care. The results from these studies show it is possible to develop a predictive model for mental health and psychiatry using clinical data. As the search for more preventative measures that allow for timely intervention strategies continues, early warning systems and risk prediction tools have the potential to mitigate the continuous deterioration. Furthermore, the findings of a study in 2014 assessing the use of modified early warning scores (MEWSs) in mental health inpatient settings suggest that the use of similar tools would be useful for providers to recognize patient deterioration and therefore act accordingly after receiving the score [35].

### Challenges and considerations

As ML becomes more widely used and incorporated into areas such as healthcare, it is important to address the ethical considerations that inherently come with the fast-evolving science of artificial intelligence. The necessity for transparency when developing models and reporting methodologies and strategies for integrating them into the healthcare system will be vital for all future studies and implementation strategies. Furthermore, patient safety and privacy should remain a priority throughout all stages of the development and future piloting of such tools.

As a consideration, we began our comprehensive search for early warning systems, trigger tools, and reporting signals for patient deterioration in psychiatric and mental health settings. While analyzing the relevant literature, we found that most of the included studies were centered around predictive tools and adverse events. Therefore, we narrowed our review on these topics.

### Limitations and future directives

While this comprehensive review of the studies contributes to providing insight and understanding on the development of predictive tools, particularly in a psychiatric setting, some limitations still exist.

This study provides a detailed review of the current literature and existing predictive models of adverse events risk in psychiatry wards. We described the different models used in each included study, including the model type, variables used, and outcomes of interest. However, there are several limitations to our study. First, the scope of the review was intentionally broad. At present, there are a limited number of studies that have used ML on routinely collected patient data to predict the occurrence of specific adverse events. Overall, there was a high degree of heterogeneity between the included studies, hindering the direct comparability of model performance. Many researchers still use traditional statistical models such as regression to predict the outcomes of interest. Finally, one challenge and limitation was the language restrictions in our literature search phase. We acknowledge that some institutions around the world have done work on this topic, some of which may have been missed due to the non-English studies exclusion criteria in place.

The few studies that used ML were in their early and initial phases, and the models did not undergo significant internal or external validation using other datasets. It may be early to determine the significance of the performance of these models, which points to future directions for further model development, testing, and validation. Future studies should consider a standardization of methodologies that may involve the inclusion of internal validation. External validation should also be considered in future piloting and implementation studies to assess the generalizability of such tools. An example of an external validation study is the use of qSOFA for patients with sepsis admitted to ICSs by comparing its AUROC score with that of SIRS [36]. In addition to validation studies, long-term implementation of these types of predictive scores and tools should be conducted to determine the feasibility and efficacy of the tool. These types of studies are important for understanding how to best integrate predictive tools into different psychiatric settings and determine what changes could be made. The implementation and deployment of such tools could potentially reduce the physical and mental risks for healthcare workers for both parties. Therefore, we suggest future studies also focus on the piloting of these tools to assess the models with real-time patients, compare the model intervention group to control groups, and assess if the predictive models provide an added benefit to key users such as clinicians.

Given the vulnerability of the patient population and the high incidence of adverse events in psychiatry, the implementation of these tools in the clinical setting needs to consider issues such as feasibility, clinician acceptability, patient privacy and safety, and ethics in addition to model performance. Ultimately, the goal of these predictive models is to support the clinician’s assessment and decision making and reduce the incidence of adverse outcomes/events.

## Supplementary Information

Supplementary Material 1.

## Data Availability

Not applicable.

## Abbreviations

ML: Machine Learning
AUC: Area under the curve
ROC: Receiver Operating Characteristic
AE: Adverse events
ICUs: Intensive Care Units

## Data availability

No datasets were generated or analysed during the current study.

## References

[1] Canadian Institute for Health Information. Patient harm in Canadian hospitals? It does happen. Available from: https://www.cihi.ca/en/patient-harm-in-canadian-hospitals-it-does-happen. Cited 2024 Feb 13.

[2] Waddell AE, Gratzer D. Patient Safety and Mental Health-A Growing Quality Gap in Canada. Can J Psychiatry. 2022;67(4):246–9. 34378413 10.1177/07067437211036596 PMC9099078

[3] Griffin F, Resar R. IHI global trigger tool for measuring adverse events (Second Edition). IHI Innovation Series white paper Cambridge, MA: Institute for Healthcare Improvement. 2009; Available from: (Available at ihi.org).

[4] D’Lima D, Crawford MJ, Darzi A, Archer S. Patient safety and quality of care in mental health: a world of its own? BJPsych Bull. 2017;41(5):241–3. 29018546 10.1192/pb.bp.116.055327 PMC5623880

[5] Kelly EL, Fenwick K, Brekke JS, Novaco RW. Well-Being and Safety Among Inpatient Psychiatric Staff: The Impact of Conflict, Assault, and Stress Reactivity. Adm Policy Ment Health. 2016;43(5):703–16. 26377816 10.1007/s10488-015-0683-4 PMC4794422

[6] Hui A, Middleton H, Völlm B. The uses of coercive measures in forensic psychiatry: a literature review. In: Völlm B, Nedopil N, editors. The use of coercive measures in forensic psychiatric care. Cham: Springer International Publishing; 2016. p. 151–84. Available from: http://link.springer.com/10.1007/978-3-319-26748-7_9. Cited 2024 Jan 22.

[7] Frueh BC, Knapp RG, Cusack KJ, Grubaugh AL, Sauvageot JA, Cousins VC, et al. Special Section on Seclusion and Restraint: Patients’ Reports of Traumatic or Harmful Experiences Within the Psychiatric Setting. PS. 2005;56(9):1123–33. 10.1176/appi.ps.56.9.1123 16148328

[8] Dumais A, Larue C, Drapeau A, Ménard G, Giguère AM. Prevalence and correlates of seclusion with or without restraint in a Canadian psychiatric hospital: a 2-year retrospective audit. J Psychiatr Ment Health Nurs. 2011;18(5):394–402. 21539684 10.1111/j.1365-2850.2010.01679.x

[9] Parmigiani G, Barchielli B, Casale S, Mancini T, Ferracuti S. The impact of machine learning in predicting risk of violence: A systematic review. Front Psychiatry. 2022;13:1015914. 36532168 10.3389/fpsyt.2022.1015914 PMC9751313

[10] Iozzino L, Ferrari C, Large M, Nielssen O, de Girolamo G. Prevalence and Risk Factors of Violence by Psychiatric Acute Inpatients: A Systematic Review and Meta-Analysis. PLoS ONE. 2015;10(6): e0128536. 26061796 10.1371/journal.pone.0128536 PMC4464653

[11] Murphy DR, Meyer AN, Sittig DF, Meeks DW, Thomas EJ, Singh H. Application of electronic trigger tools to identify targets for improving diagnostic safety. BMJ Qual Saf. 2019;28(2):151–9. 30291180 10.1136/bmjqs-2018-008086 PMC6365920

[12] Fazel S, Toynbee M, Ryland H, Vazquez-Montes M, Al-Taiar H, Wolf A, et al. Modifiable risk factors for inpatient violence in psychiatric hospital: prospective study and prediction model. Psychol Med. 2023;53(2):590–6. 34024292 10.1017/S0033291721002063 PMC9899559

[13] Ogonah MGT, Seyedsalehi A, Whiting D, Fazel S. Violence risk assessment instruments in forensic psychiatric populations: a systematic review and meta-analysis. Lancet Psychiatry. 2023;10(10):780–9. 37739584 10.1016/S2215-0366(23)00256-0 PMC10914679

[14] Li Y, Pu Q, Li S, Zhang H, Wang X, Yao H, et al. Machine learning methods for research highlight prediction in biomedical effects of nanomaterial application. Pattern Recogn Lett. 2019;117:111–8.

[15] Wang Y, Liu L, Wang C. Trends in using deep learning algorithms in biomedical prediction systems. Front Neurosci. 2023;17:1256351. 38027475 10.3389/fnins.2023.1256351 PMC10665494

[16] Hosein F, Bobrovitz N, Berthelot S, Zygun D, Ghali WA, Stelfox HT. A systematic review of tools for predicting severe adverse events following patient discharge from intensive care units. Crit Care. 2013;17(3):R102. 23718698 10.1186/cc12747 PMC4056089

[17] Qiu X, Lei YP, Zhou RX. SIRS, SOFA, qSOFA, and NEWS in the diagnosis of sepsis and prediction of adverse outcomes: a systematic review and meta-analysis. Expert Rev Anti Infect Ther. 2023 3;21(8):891–900. 37450490 10.1080/14787210.2023.2237192

[18] NHS. NHS England. National Early Warning Score (NEWS). Available from: NEWS2 has seen widespread uptake across the NHS in England – at present 100% of ambulance trusts and 76% of acute trusts are using NEWS2, with other early warning scores in place in other areas. 2017.

[19] Welch J, Dean J, Hartin J. Using NEWS2: an essential component of reliable clinical assessment. Clin Med (Lond). 2022;22(6):509–13. 36427875 10.7861/clinmed.2022-0435 PMC9761428

[20] Garrubba M, Joseph C. Recognising and responding to deterioration in mental state: A scoping review. Centre for Clinical Effectiveness, Monash Health: Melbourne, Australia; 2019.

[21] Rice ME, Harris GT, Lang C. Validation of and revision to the VRAG and SORAG: The Violence Risk Appraisal Guide—Revised (VRAG-R). Psychol Assess. 2013;25(3):951–65. 23647040 10.1037/a0032878

[22] Madakam S, Uchiya T, Mark S, Lurie Y. Artificial Intelligence, Machine Learning and Deep Learning (Literature: Review and Metrics). Asia-Pacific Journal of Management Research and Innovation. 2022;18(1–2):7–23.

[23] Pou-Prom C, Murray J, Kuzulugil S, Mamdani M, Verma AA. From compute to care: Lessons learned from deploying an early warning system into clinical practice. Front Digit Health. 2022;4: 932123. 36133802 10.3389/fdgth.2022.932123 PMC9483018

[24] Verma AA, Murray J, Greiner R, Cohen JP, Shojania KG, Ghassemi M, et al. Implementing machine learning in medicine. CMAJ. 2021 30;193(34):E1351–7. 35213323 10.1503/cmaj.202434 PMC8432320

[25] Tricco AC, Lillie E, Zarin W, O’Brien KK, Colquhoun H, Levac D, et al. PRISMA extension for scoping reviews (PRISMA-ScR): checklist and explanation. Ann Intern Med. 2018;169(7):467–73. 30178033 10.7326/M18-0850

[26] Chapter 11: Scoping reviews. In: JBI Manual for Evidence Synthesis. JBI; 2020. Available from: https://jbi-global-wiki.refined.site/space/MANUAL/4687342/Chapter+11%3A+Scoping+reviews. Cited 2024 Jan 19.

[27] Menger V, Scheepers F, Spruit M. Comparing Deep Learning and Classical Machine Learning Approaches for Predicting Inpatient Violence Incidents from Clinical Text. Appl Sci. 2018;8(6):981.

[28] Gou N, Xiang Y, Zhou J, Zhang S, Zhong S, Lu J, et al. Identification of violent patients with schizophrenia using a hybrid machine learning approach at the individual level. Psychiatry Res. 2021;306: 114294. 34823086 10.1016/j.psychres.2021.114294

[29] Menger V, Spruit M, van Est R, Nap E, Scheepers F. Machine Learning Approach to Inpatient Violence Risk Assessment Using Routinely Collected Clinical Notes in Electronic Health Records. JAMA Netw Open. 2019 3;2(7): e196709. 31268542 10.1001/jamanetworkopen.2019.6709 PMC6613290

[30] Suchting R, Green CE, Glazier SM, Lane SD. A data science approach to predicting patient aggressive events in a psychiatric hospital. Psychiatry Res. 2018;268:217–22. 30064068 10.1016/j.psychres.2018.07.004

[31] Danielsen AA, Fenger MHJ, Østergaard SD, Nielbo KL, Mors O. Predicting mechanical restraint of psychiatric inpatients by applying machine learning on electronic health data. Acta Psychiatr Scand. 2019;140(2):147–57. 31209866 10.1111/acps.13061

[32] Wang KZ, Bani-Fatemi A, Adanty C, Harripaul R, Griffiths J, Kolla N, et al. Prediction of physical violence in schizophrenia with machine learning algorithms. Psychiatry Res. 2020;289: 112960. 32361562 10.1016/j.psychres.2020.112960

[33] Greytak R, Wang JY, Hsu YJ, Marsteller J, Jayaram G. Use of Rapid Response Teams in Psychiatry: Variables that Impact Safety. J Psychiatr Pract. 2017;23(6):390–400. 29303946 10.1097/PRA.0000000000000270

[34] Geiss M, Chamberlain J, Weaver T, McCormick C, Raufer A, Scoggins L, et al. Diagnostic Overshadowing of the Psychiatric Population in the Emergency Department: Physiological Factors Identified for an Early Warning System. J Am Psychiatr Nurses Assoc. 2018;24(4):327–31. 28862084 10.1177/1078390317728775

[35] Shaddel F, Khosla V, Banerjee S. Effects of introducing MEWS on nursing staff in mental health inpatient settings. Prog Neurol Psychiatry. 2014;18(2):24–7.

[36] April MD, Aguirre J, Tannenbaum LI, Moore T, Pingree A, Thaxton RE, et al. Sepsis Clinical Criteria in Emergency Department Patients Admitted to an Intensive Care Unit: An External Validation Study of Quick Sequential Organ Failure Assessment. J Emerg Med. 2017;52(5):622–31. 27823893 10.1016/j.jemermed.2016.10.012

