# Supplementary material for: The development of early warning scores or alerting systems for the prediction of adverse events in psychiatric patients: a scoping review
Source: BMC Psychiatry. 2024 Oct 28;24:742. doi: 10.1186/s12888-024-06052-z (PMC11520586; doi:10.1186/s12888-024-06052-z)
Supplement: Supplementary file 1 — Supplementary Material 1. [file 12888_2024_6052_MOESM1_ESM.docx]

**Table 1. Summary of studies included in the review (n = 9)**

| **Author, Year (citation)** | **Study Design** | **Journal** | **Country** | **Population Diagnoses (Main Disorder)** | **Setting** | **Sample size** | **Type of Adverse Events/Outcome** | **Patient Characteristics (gender, primary diagnosis)** | **Average age [Standard Deviation] (in years)** | **Risk Prediction Model** |
| --- | --- | --- | --- | --- | --- | --- | --- | --- | --- | --- |
| Danielsen et al., 2019 (1) | Retrospective cohort/Data linkage study/ | Acta Psychiatrica Scandinavica | Denmark | Adult patients with mental disorders (some with multiple mental disorders and/or comorbid substance use disorder) | Psychiatric departments in the Central Denmark Region (CDR) | 5050 patients, 8869 admissions | Incidents of mechanical restraint | 51% male; 49% female  42% without any prior mental disorder diagnosis; 24% prior mood disorder; 11% psychotic disorders |  | ML: Supervised machine learning algorithms  They trained five models (neural network, support vector machine, random forest, stepwise forward logistic regression, LASSO).  They used the best performing model for validation using the test set. |
| Fazel et al., 2021 (2) | Prospective cohort study | Psychological Medicine | United Kingdom | Adult psychiatric inpatients in forensic and general wards in psychiatric hospitals | Three psychiatric hospitals within one UK National Health Service organisation.  Eight forensic wards (79 beds) and two general adult wards (43 wards). | 89 inpatients | Violent incidents categorised as ‘Violence’ or ‘aggression’ incidents by the Datix incident reporting system. | 80% male patients; 20% female patients  70% with a primary diagnosis of schizophrenia-spectrum disorder; 20% with a primary diagnosis of personality disorder | 38.9 (12.5) | Non-ML:  Multilevel logistic regression model (using patient as a random effect).  This model assessed the association between total dynamic score and the occurrence of violent incidents. |
| Geiss et al., 2018 (3) | Retrospective cohort study | Journal of the American Psychiatric Nurses Association | United States | Patients admitted to the psychiatric unit | Psychiatric unit in a 500-bed acute care hospital. | 231 Medical records (83 records in cohort A; 148 in cohort B).  133 psychiatric admissions | Medical emergent event (in patients with a psychiatric presentation) | Experimental group = 48.19%.  Control group = 45.27%.  In the experimental group were admitted patients who experienced an emergent medical event occur  during length of stay.  In the control group were admitted patients who did not experience an emergent medical event. | Experimental = 59  Control group = 46 | Non-ML: Logistic regression |
| Gou et al., 2021 (4) | Retrospective and Multimodal | Psychiatry Research | China | Adult, male patients with schizophrenia with violent offenses and patients without violent offenses from the general psychiatric ward.  Participants with schizophrenia who were violent offenders were recruited from November 2011 to November 2020 from the  forensic psychiatry department of the Second Xiangya Hospital of Central South University.  Non-violent patients were recruited from the general psychiatric wards. | From a forensic and general psychiatry department at the Second Xiangya Hospital of Central South University. | Total participants n = 74.  Participants with schizophrenia who were violent offenders (VSZ) (n = 42).  Non-violent patients with schizophrenia (NVSZ) (n = 32). | The risk of violence in patients (violent crimes, including killing or assaulting other people). | All male (inclusion criteria).  All participants had a diagnosis of schizophrenia confirmed using the International Classification of Diseases Version 10 (ICD-10). | Participants VSZ = 30.88 (7.17).  NVSZ = 28.00 (6.09). | ML: Least Absolute Shrinkage and Selection Operator (LASSO) regression and Support Vector Machine |
| Greytak et al., 2017 (5) | Retrospective chart review (matched case-control) | Journal of Psychiatric Practice | United States | Patients admitted to inpatient psychiatric units.  General inpatients and specialty psychiatric services | The Johns Hopkins Hospital Department of Adult Inpatient Psychiatry | 180 (60 cases and 120 controls) | Medical deterioration leading to emergent medical intervention (full code or rapid response) | Cases group = 48.3%  Control group = 46.7% | Cases group = 52.4 (range: 37.5-68.5).  Control group = 52.7 (range: 36.5-71). | Non-ML: Multiple logistic regression using forward stepwise regression |
| Menger et al., 2018 (6) | Experimental evaluation of machine learning techniques (deep learning vs. classical ML)  Retrospective | Applied sciences | The Netherlands | Psychiatric patients admitted to all six inpatient units (includes child, adolescent, and adult patients). | The Psychiatry Department of the University Medical Center Utrecht. | 2521 admissions from 1796 patients | Violent behaviour and incidents (directed at staff or at other patients, including verbal and physical) |  |  | Deep learning model: document embeddings (paragraph2vec) with a Recurrent Neural Network |
| Menger et al., 2019 (7) | Prognostic study | JAMA Network Open | Netherlands | Patient data was collected from 2 independent psychiatric treatment centers.  Diagnoses included Anxiety disorder,  Bipolar disorder,  Delirium, dementia, amnesia, and other cognitive disorders,  Depressive disorder,  Developmental disorder,  Eating disorder,  Mood disorder,  Personality disorder,  Substance-related disorder,  Schizophrenia, or other psychotic disorder. | Psychiatric centres | 4128  (2209 at site 1)  (1919 at site 2) | Inpatient violent incidents.  A mandatory reporting of all violent incidents (patient-staff and patient-patient violence) on the incident form. The severity of the incident was measured using the Staff Observation Aggression Scale–Revised.  Violent incidents included all threatening and violent behavior (verbal or physical) directed at another person but excluded self-harm and inappropriate behavior, such as substance use, sexual intimidation, or vandalism. | Site 1: 48.2% male  Site 2: 64.5% male | Site 1: 34.0 [16.6]  Site 2:  45.9 [16.6] | ML:  Paragraph2vec algorithm which learns an accurate numerical representation from a large corpus of text in an unsupervised way.  This study used retrospective textual data, to train the model to differentiates patients who show violent behavior during the first 4 weeks of admission from patients who do not. |
| Suchting et al., 2018 (8) | Retrospective study | Psychiatry Research | United States (Texas) |  | Harris County Psychiatric Center. | 29,841 patients | An aggressive event toward staff or patient. | 57.1% males | 33.3 years (13.7) | ML:  This study used four ML algorithms,  Penalized generalized linear modeling (GLM), random forest (RF), gradient boosting machine (GBM), and deep neural networks (DNN). |
| Wang et al., 2020 (9) | Retrospective cross-sectional study | Psychiatry Research | Canada | Solely patients with schizophrenia diagnosis. | Canadian Mental Health Association (CAMH). | 275 schizophrenia patients. | Severity scores ranging from an absence of physical violence to assault resulting in serious bodily injury, as outlined in the Modified Overt Aggression Scale (MOAS) | Through EMR, 103 patients were identified as violent (78.64% males) and 172 were identified as non-violent (64.44% males).  On average, 71.64% of patients were males. | Violent patients: 44.82 years (12.95).  Non-violent patients: 38.54 years (12.83). | This study used one multivariable statistical method (binary logistic regression model) and six ML classification algorithms for model prediction comparison: the least absolute shrinkage and selection operator (lasso), elastic net, random forest, gradient boosted regression trees (GBRT), support vector machine (SVM) classifier, and SVM classifiers with radial basis function (RBF) kernels |

**Table 2. Performance Measures of Predictive Models (Primary Outcome)**

| **Author and year (citation)** | **Outcome predicted/predicted outcome** | **Data source** | **Selected predictive model** | **Validation (if yes, describe)** | **Performance of the selected model** | **Validity of the selected model (if validity was completed)** | **Selected (strongest) predictor variables** | **Limitations** |
| --- | --- | --- | --- | --- | --- | --- | --- | --- |
| Danielsen et al., 2019 (1) | Incident of mechanical restraint episode occurring between the first hour of admission and the first 3 days following admission. | Electronic health data (clinical notes in natural language, binary data, numerical data) | Supervised Machine Learning (Random Forest algorithm) | Validated using an independent test dataset.  They set aside 70% of admissions for the training set and 30% of admissions for the test set (used for validation). | - AUC of 0.87 (95% CI 0.79-0.93) | - At 94% specificity, sensitivity was 56% and PPV was 8.1% - At 88% specificity, sensitivity was 74% and PPV was 6.0% | - 45 predictors were included in the final Random Forest model - The top 10 predictors ranked by importance (see Table 3) | - The risk of MR recurrence was not considered, only incident episodes or MR. |
| Fazel et al., 2021(2) | Aggression and interpersonal violence as recorded on the incident reporting system | Dynamic variables: web-based risk monitoring tool (an online questionnaire with 10 questions, each with a 5-point Likert scale assessing the patient’s dynamic risk factors)  Static variables: patient charts | Multilevel logistic regression (in a fixed-effect model using all covariates) | Model needs further internal validation (i.e., resampling techniques like boot-strapping or cross-validation) as well as validation using independent external samples | - AUC of 0.77 (95% CI: 0.72-0.82) with inclusion of the dynamic scores and 0.75 (0.70-0.80) without the dynamic scores |  | The main model included the following variables: total dynamic score as a binary variable (>0 vs. 0), age, female sex, ward (forensic vs. general), and diagnosis. A total dynamic score greater than 0, female sex, and younger age were associated with an increased risk of violent incidents.  None of the ten dynamic items were predictive on their own. | The risk factors were identified from a large systemic review and meta-analysis based on outpatient and inpatient settings.  Outcome data was based on routinely collected data which is likely underreported. |
| Geiss et al., 2018 (3) | Emergent medical event | Medical records | Bivariate chi-square tests were first performed to determine which of the 17 variables were significant to be included in a logistic regression model. | No | - Sensitivity of the screening tool was 68.7% and specificity was 85.5% when the total score was greater than 2 (i.e., if two or more predictive factors were present) |  | Predictive variables used to create a new screening tool: advanced age (greater than 70 years), abnormal heart rate (below 60 or above 100), abnormal temperature (38 degrees Celsius or higher) | Small sample size and likely residual confounding to explain the differences in the two comparison groups |
| Gou et al., 2021 (4) | Violent offenses (violent crimes, including killing or assaulting other people), and degree of violence as quantified by Modified Overt Aggression Scale scores | Sociodemographic-clinical features (clinical data) and three modalities of neuroimaging data | ML: Least Absolute Shrinkage and Selection Operator (LASSO) regression and Support Vector Machine | No external validation | - Socio-demographic and clinical model (with four variables): AUC of 0.91, sensitivity of 75.76% and specificity of 83.33% - 3-way neuroimaging data: AUC of 0.91, sensitivity of 93.94%, specificity of 80.95% - Combined neuroimaging and sociodemographic data: AUC of 0.95, sensitivity of 90.91%, specificity of 90.48% |  | Discriminative clinical variables included hostility, psychopathy, low education, and overall score on the HCR-20 (violence risk assessment).  Discriminative neuroimaging features: frontotemporal pathway and the subcortical system (i.e., temporal, frontal, parietal, and striatum systems). | Strict inclusion criteria (only included male patients without any other comorbidities, including substance abuse); limited information on the patient’s history of medication status, unable to study the effect of substance abuse; small sample size |
| Greytak et al., 2017 (5) | Use of emergent medical intervention (e.g., psychiatric inpatients requiring a rapid response or full code) | Patient charts in electronic health records | Multiple logistic regression models using the forward stepwise selection of variables | Not known | - 12-variable model: AUC of 0.91 and a McFadden pseudo-R^2 of 47.5% - 6-variable “SCHEME”model: AUC of 0.76 and R^2 of 19.1% |  | Six-item “SCHEME” Model: history of stenting/angioplasty, chronic psychotic illness, hepatitis C, history of epilepsy, history of mood disorder, and electrolyte abnormality | Most variables were measured in a binary manner (present/not present) and do not reflect acuity or severity.  Single site and small sample size. |
| Menger et al., 2018 (6) | Patient violence incident occurring in the first 30 days of admission | Clinical texts from EHR | Deep learning compared to classical ML | Not known | Best prediction obtained by combining Document Embeddings (paragraph2vec) with a Recurrent Neural Network: AUC of 0.788 ±0.018 |  | Clinical text was used for assessing violence risk, significant text variables were not specified | Potential overfitting from testing several combinations of text representations and classification models; only used unstructured clinical text for prediction (no use of structured variables like medication use, diagnosis, and patient demographics); moderate sample size |
| Menger et al., 2019 (7) | Inpatient violence risk from clinical notes written in patients’ electronic health records. | Electronic health data (clinical notes in natural language) | ML Paragraph2vec algorithm (deep learning-based) which learns an accurate numerical representation from a large corpus of text in an unsupervised way.  A multivariable prognostic model. | Nested cross- validation used to train and evaluate models that assess violence risk during the first 4 weeks of admission based on clinical notes available after 24 hours. | - Predictive validity:   AUC at site 1 (AUC = 0.797; 95% CI, 0.771-0.822) and site 2 (AUC = 0.764; 95% CI, 0.732-0.797) | - The validation of pretrained models in the other site:  AUC = 0.722 (95% CI, 0.690-0.753) at site 1. AUC = 0.643 (95% CI, 0.610-0.675) at site 2 - The difference in AUCs between the internally trained model and the model trained on other-site data was significant at site 1 (AUC difference = 0.075; 95% CI, 0.045-0.105; P < .001) and site 2 (AUC difference = 0.121; 95% CI, 0.085-0.156; P < .001). | Internally validated predictions resulted in AUC values with good predictive validity. | The validation of trained models using data from other sites suggests that violence risk assessment generalizes modestly to different populations.  The data obtained from EHRs was intended for treatment plan rather than research which introduced noise in the data.  Since this study used a black box approach, the terms selected, and the subgroup analysis did not directly explain the model behavior. |
| Suchting et al., 2018 (8) | The presence or absence of an aggressive event toward staff or patients. | Electronic medical health records. | The best performing algorithm is the Penalized generalized linear modeling (GLM)  Other models tested were random forest (RF), gradient boosting machine (GBM), and deep neural network (DNN). | 5-fold cross validation modeling using the other algorithms used.  The cross-validated AUC of the tuned (best-fitting) model for each algorithm | - Algorithm training AUC = 0.7794 | - Algorithm testing AUC = 0.7801 | Initially, the final set of variables derived included 328 predictor variables (313 categorical, 15 continuous).  The GLM retained 75 variables. The top 20 variables can be seen in Table 3. | The data was taken from a single inpatient facility limiting the generalizability of the algorithm to other psychiatric facilities. |
| Wang et al., 2020 (9) | The presence of violence. | Retrospective review of patient electronic medical records. | The best performing model was the random forest models.  The other algorithms included were binary logistic regression model, the least absolute shrinkage and selection operator (lasso), elastic net, gradient boosted regression trees (GBRT), support vector machine (SVM) classifier, and SVM classifiers with radial basis function (RBF) kernels. | This study used a stratified k-fold cross validation technique (k = 5).  The sample population was divided into five equally sized subgroups (folds). They performed five rounds of training and validation. | Random Forests Model:   - Area under the receiver operator characteristic curve (AUROC) = 0.63 - Accuracy = 0.62 - Sensitivity = 0.32 - Specificity = 0.80 - Positive predictive value (PPV) = 0.62 - Negative predictive value (NPV) = 0.54 - P-value < 0.001 |  | In this study, 28 predictive variables were derived. | Since this study had seven models, it could have benefited from a larger sample size and another psychiatric facility/centre to train validate the models. |

**Table 3: Variables Included in the Model (ML or non-ML) (Secondary Outcomes)**

| **Author and Year (citation)** | **Models (*selected model)** | **Variables included in the selected/best performing model** |
| --- | --- | --- |
| Danielsen et al., 2019 (1) | Supervised machine learning algorithms (neural network, support vector machine, random forest*, stepwise forward logistic regression, LASSO) | The final random forest model included 45 predictors. The top 10 predictors ranked by relative importance are listed below:   - Admission type [categorical] (voluntary, involuntary because of danger, involuntary because of urgent need for treatment) - Broset Violence Checklist [categorical] (total sum of zero, total sum greater than zero, missing value)   The other 8 were derived from clinical notes (in natural language) in the EHR with seven belonging to the theme “Subjective Mental State” and one belonging to the theme “Current Objective Mental State”   - Somatic comorbidity - Sparse/non-coherent verbal response - Non-informative verbal response - Abnormal behaviour (predominantly male patients) - Threatening behaviour - Good social status - Suicidal ideation - specifically involving car crash - Persecutory ideation |
| Fazel et a., 2021 (2) | Multilevel logistic regression (in a fixed-effect model using all covariates) | Total dynamic score as a binary variable (>0 vs. 0), age (per increase in 10 years), sex (female vs. male), ward (forensic vs. general), and diagnosis (schizophrenia-spectrum disorder vs. other diagnoses).  The study used 10 dynamic scores from assessments (modifiable variables):   - Non-adherence with therapy - Non-adherence with medication - Aggression (verbal or physical) - Discharge of tension and emotions - Emergence/deterioration of paranoid/persecutory delusions - Emergence/deterioration of hallucinations - Increasing anger due to psychotic symptoms - Emergence/increase in drug misuse - Emergence/increase in alcohol misuse - Increase in anxiety |
| Geiss et al., 2018 (3) | Logistic regression model | Advanced age (greater than 70 years), abnormal heart rate (below 60 or above 100), abnormal temperature (38 degrees Celsius or higher) |
| Gou et al., 2021 (4) | ML: Least Absolute Shrinkage and Selection Operator (LASSO) regression and Support Vector Machine | Discriminative clinical variables included hostility, psychopathy, low education, and overall score on the HCR-20 (violence risk assessment).  Discriminative neuroimaging features: frontotemporal pathway and the subcortical system (i.e., temporal, frontal, parietal, and striatum systems). |
| Greytak et al., 2017 (5) | Multiple logistic regression models using the forward stepwise selection of variables | The most exhaustive model from a stepwise forward regression yielded the following 12 significant variables: positive toxicology screen, total number of medications, chronic psychotic illness, epilepsy, history of angioplasty/stenting, cancer, electrolyte abnormality, voluntary, hepatitis C, sex, mood disorder, history of pulmonary embolism.  A 6-variable model with good predictive power was then created to generate a simple screening tool.  The six-item “SCHEME” model includes the following variables: history of stenting/angioplasty, chronic psychotic illness, hepatitis C, epilepsy, history of mood disorder, and electrolyte abnormality |
| Menger et al., 2018 (6) | Classical ML models: Neural Networks, Bayesian Classifiers, Support Vector Machines, and Decision Trees  Novel Deep Learning techniques: Recurrent Neural Networks, Convolutional Neural Network  Each classification model was combined with one of the following text representations: bag-of-words binary, bag-of-words tf-idf, word embeddings (word2vec), document embeddings (paragraph2vec) | Best prediction obtained by combining Document Embeddings (paragraph2vec) with a Recurrent Neural Network  Clinical text was used as the data source. Significant text variables were not specified. |
| Menger et al., 2019 (7) | Paragraph2vec algorithm which learns an accurate numerical representation from a large corpus of text in an unsupervised way.  This study used retrospective textual data, to train the model to differentiates patients who show violent behavior during the first 4 weeks of admission from patients who do not. | Demographic variables were limited to sex, year of birth, and Diagnostic and Statistical Manual of Mental Disorders (Fourth Edition) diagnosis.  Of the 1000 most frequent terms from clinical notes, the top 20 terms by generalizability within a data set were selected:  Site 1:   - Agressief (aggressive) - Reageert (reacts) - Aangeboden (offered) - Boos (angry) - Deur (door) - Loopt (walks) - Ibs (arrest) - Aanbieden (offer) - Noodmedicatie (emergency medication) - Liep (walked) - Agressie (aggression) - Vraagt (asks) - Status vrijwillig (status voluntary) - Psychotisch (psychotic) - Collega (colleague) - Spreekt (speaks) - Gehouden (obliged) - Beoordelen ( judge), verb - Momenten (moments) - Somber (dejected)   Site 2:   - Verbaal (verbal) - Dreigend (threatening) - Agressie (aggression) - Hierop ([up]on this) - Kantoor (office) - Personeel (staff) - Aangesproken (spoke to) - Agressief (aggressive) - Gevaar agressie (danger aggression) - Agitatie (agitation) - Geirriteerd (irritated) - Separeer (seclusion room) - Loopt (walks) - Grond (ground) - Aanvang (commencement) - Mede (also) - Dhr wilde (Mr wanted) - Liep (walked) - Geagiteerd (agitated) - cvd (not available) |
| Suchting et al., 2018 (8) | Penalized generalized linear modeling (GLM) - supervised ML | Top 20 predictor variables of the 75 that the GLM model retained:   - Current Living Situation (Homeless) - Legal History - Assault Conviction (Yes) - Abuse History - Witnessed (Other) - Abuse History - Perpetrated (Other) - Age - Risk Assessment - Aggression History (No) Education History - High School Graduate (Other) Admission - Positive Suicidal Ideation (Yes) Education History - Special Classes (Yes) - ADRS - Depression - Work History (Problematic) - Marital History - Has Children (No) - Sleep - Requires Relaxation Aids (Yes) - Risk Assessment - Family History of Suicide (Other) Marital History - Currently Married (Single, Never) Mental Status - Logical/Goal Directed (No) - Risk Assessment - Safety Contract (Other) - Financial - Medicaid (Yes) - Mental Status - Logical/Goal Directed (Yes) - Work History (No Work Experience) |
| Wang et al., 2020 (9) | Supervised ML: Random Forest Algorithm | - Age (μ ± s.d.) - Sex (male/ female) - European caucasian - English as primary language - Religious identity - Immigrated before age 9 - Immigrated between age 10 and 18 - Immigrated after age 18 - Age-of-onset (μ ± s.d.) - Number of hospitalizations (N > 5) - Suicide attempter - Lifetime alcohol abuse/ dependence - Lifetime marijuana abuse/ dependence - Lifetime drug abuse/ dependence - Family history of psychosis - Family history of mood disorders - Family history of suicidal behavior - NEO neuroticism (μ ± s.d.) - NEO extraversion (μ ± s.d) - NEO openness (μ ± s.d) - NEO agreeableness (μ ± s.d) - NEO conscientiousness (μ ± s.d) - CTQ physical abuse (μ ± s.d) - CTQ emotional abuse (μ ± s.d) - CTQ sexual abuse (μ ± s.d) - CTQ physical neglect (μ ± s.d) - CTQ emotional neglect (μ ± s.d) - CTQ total score (μ ± s.d.) |

**References**

1. Danielsen AA, Fenger MHJ, Østergaard SD, Nielbo KL, Mors O. Predicting mechanical restraint of psychiatric inpatients by applying machine learning on electronic health data. Acta Psychiatrica Scandinavica. 2019;140(2):147–57.

2. Fazel S, Toynbee M, Ryland H, Vazquez-Montes M, Al-Taiar H, Wolf A, et al. Modifiable risk factors for inpatient violence in psychiatric hospital: prospective study and prediction model. Psychol Med. 2023 Jan;53(2):590–6.

3. Geiss M, Chamberlain J, Weaver T, McCormick C, Raufer A, Scoggins L, et al. Diagnostic Overshadowing of the Psychiatric Population in the Emergency Department: Physiological Factors Identified for an Early Warning System. J Am Psychiatr Nurses Assoc. 2018;24(4):327–31.

4. Gou N, Xiang Y, Zhou J, Zhang S, Zhong S, Lu J, et al. Identification of violent patients with schizophrenia using a hybrid machine learning approach at the individual level. Psychiatry Res. 2021 Dec;306:114294.

5. Greytak R, Wang JY, Hsu YJ, Marsteller J, Jayaram G. Use of Rapid Response Teams in Psychiatry: Variables that Impact Safety. J Psychiatr Pract. 2017 Nov;23(6):390–400.

6. Menger V, Scheepers F, Spruit M. Comparing Deep Learning and Classical Machine Learning Approaches for Predicting Inpatient Violence Incidents from Clinical Text. Applied Sciences. 2018 Jun;8(6):981.

7. Menger V, Spruit M, van Est R, Nap E, Scheepers F. Machine Learning Approach to Inpatient Violence Risk Assessment Using Routinely Collected Clinical Notes in Electronic Health Records. JAMA Netw Open. 2019 Jul 3;2(7):e196709.

8. Suchting R, Green CE, Glazier SM, Lane SD. A data science approach to predicting patient aggressive events in a psychiatric hospital. Psychiatry Res. 2018 Oct;268:217–22.

9. Wang KZ, Bani-Fatemi A, Adanty C, Harripaul R, Griffiths J, Kolla N, et al. Prediction of physical violence in schizophrenia with machine learning algorithms. Psychiatry Res. 2020 Jul;289:112960.
